# Assessment of Vitamin D, Vitamin B12, and Folate Levels in Recently Identified Pregnant Females

**DOI:** 10.7759/cureus.68514

**Published:** 2024-09-03

**Authors:** Aveen Mustafa

**Affiliations:** 1 Department of Clinical Chemistry, College of Medicine, University of Duhok, Duhok, IRQ

**Keywords:** duhok, pregnancy, vitamin-d, vitamin b12, folate

## Abstract

*Background*: Pregnancy is a critical period where optimal maternal and fetal health depends on adequate nutritional status. Deficiencies in essential vitamins, such as vitamin D, vitamin B12, and folate, can result in adverse health outcomes.

*Objective*: This study aims to assess the serum levels of vitamin D, vitamin B12, and folate in early pregnancy.

*Methodology*: This cross-sectional research was conducted at Kurdistan Private Hospital in Duhok, Kurdistan Region of Iraq, from September 2022 to October 2023. The study included 150 pregnant women, with ages ranging from 18 to 45 years. Serum levels of vitamin D, vitamin B12, and folate were measured using the Automatic Clinical Chemistry Analyzer COBAS e 411 (Roche Diagnostics, Basel, Switzerland).

*Results*: The mean age of the participants was 29 years (standard deviation [SD] = 6.2 years), with ages ranging from 18 to 45 years. The average serum human chorionic gonadotropin (HCG) level was 4,292 mIU/mL (SD = 3,947 mIU/mL), with a range of 98 to 10,000 mIU/mL. The mean serum levels were 17.8 ng/mL (SD = 11.6) for vitamin D, 367 pg/mL (SD = 245) for vitamin B12, and 11.5 ng/mL (SD = 4.6) for folate. The prevalence of vitamin D deficiency was significant, with 92 participants (61.3%) having levels below 20 ng/mL, 39 participants (26%) having insufficient levels ranging from 20 to 29 ng/mL, and only 19 participants (12.7%) having sufficient levels between 30 and 100 ng/mL. No cases of vitamin D toxicity (>150 ng/mL) were observed. Regarding vitamin B12, 32 participants (21.3%) had deficient levels (<200 pg/mL), while 118 participants (78.7%) had normal levels. Folate analysis revealed that 3 participants (2%) had moderate deficiency (2-3 ng/mL), 14 participants (9.3%) had mild deficiency (3-6 ng/mL), and there were no cases of severe folate deficiency (<2 ng/mL). Pearson correlation analysis showed weak correlations between the study variables, with the strongest being a weak negative correlation between age and serum folate levels (-0.18).

*Conclusions*: The study found a high prevalence of vitamin D deficiency among the pregnant women included in the study, while the levels of folate and vitamin B12 were comparable to worldwide estimates. These findings focus attention on the importance of monitoring and addressing nutritional deficiencies at the beginning and throughout pregnancy. They also underline the need for preventive health interventions to correct these deficiencies and achieve the best outcomes for both maternal and fetal health.

## Introduction

Women of childbearing age who can become pregnant should take 0.4 mg of folic acid daily to minimize the likelihood of having a fetus with spina bifida or other neural tube defects (NTDs). Women with a history of NTD-affected pregnancies are at a higher risk of recurrence [1]. Maternal folate deficiency at the time of conception is well known to be associated with NTDs, which are congenital malformations with a significant adverse impact on the offspring’s quality of life. Preconception folate supplementation can reduce the risk of NTDs by 70% [2,3]. Vitamin B12 deficiency can adversely affect both the mother and baby by causing megaloblastic anemia and interfering with DNA synthesis. The prevalence of vitamin B12 deficiency in pregnant women worldwide is concerning and underscores the need for proper monitoring and supplementation [2,4]. Vitamin D insufficiency in pregnant women adversely impacts fetal skeletal development and is also associated with higher risks of preeclampsia, gestational diabetes, and other pregnancy complications [5,6]. Despite established preconception folic acid supplementation programs, uptake remains low in many regions. For instance, in Lebanon, only 14% of women use folic acid before pregnancy [7]. In northern China, despite a high prevalence of NTDs, awareness and usage of folic acid for NTD prevention remain inadequate, with less than 36% of women being aware of it and only 15% using it before conception [8]. Recent evaluations of a folic acid supplementation program in China indicate a significant increase in utilization among pregnant women. Rates have risen from 15% to 85% in high-prevalence areas and from 66% to 92% in low-prevalence regions [9].

In Duhok City, a premarital screening program has been implemented for the past two decades, during which mandatory folic acid supplementation (5 mg/day) is provided three months before marriage. This program ensures adequate folate levels at conception, thereby helping to prevent complications from NTDs.

This cross-sectional study aims to assess serum levels of folate, vitamin B12, and vitamin D among recently identified pregnant women in Duhok. By providing local data, we seek to assist health authorities in making informed decisions on public health strategies to promote maternal health and optimize pregnancy outcomes.

## Materials and methods

This cross-sectional study included 150 recently identified pregnant females who attended the Kurdistan Private Hospital laboratory department in Duhok, Kurdistan Region of Iraq, over one year, from September 2022 to October 2023. The study was approved by the Institutional Ethics Committee of Duhok Medical College, University of Duhok, Kurdistan Regional Government, Iraq (No. 1312F). For data collection, the participants who attended our laboratory for pregnancy testing had their diagnosis confirmed through measured β-human chorionic gonadotropin (β-HCG) levels. Following this, they were interviewed to complete detailed questionnaires about their demographic data. The aim of the study was explained to them, and informed consent was obtained from all participants before their enrollment.

Inclusion criteria were recently identified pregnant females who attended the laboratory department at Kurdistan Private Hospital. Exclusion criteria included those with a history of folic acid, vitamin B12, or vitamin D supplementation or who were currently using any vitamin supplements or medications that could interfere with vitamin absorption, such as anticonvulsants or corticosteroids, at the time of recruitment or during the previous three months. Women with existing chronic conditions that could affect vitamin levels, such as renal diseases, liver diseases, gastrointestinal disorders, or malabsorption syndromes, were also excluded.

Laboratory analysis

Approximately 5 mL of venous blood was withdrawn from the pregnant woman under aseptic conditions and was processed immediately. The blood was collected into a vacutainer tube containing a clot activator gel (AYSET-GEL) for serum. The gel tube was left to clot at room temperature for 30-40 minutes. After clotting, the serum was separated by centrifugation at 3,500 rpm for 10 minutes. The blood samples were then processed immediately by measuring the vitamin D, vitamin B12, and folate levels using chemiluminescent immunoassay techniques with the Automatic Clinical Chemistry Analyzer COBAS e 411 (Roche Diagnostics, Basel, Switzerland).

My apologies for the confusion. Here is the revised version without em dashes and with consistent capitalization:

The reference ranges are as follows: vitamin D levels (deficiency, < 20 ng/mL; insufficiency, 20-29 ng/mL; sufficiency, 30-100 ng/mL; toxicity, >150 ng/mL) [10]; vitamin B12 levels (deficiency, <200 pg/mL; normal, 200-900 pg/mL) [11]; and serum folate levels (normal, > 6 ng/mL; mild deficiency, 3-6 ng/mL; moderate deficiency, 2-3 ng/mL; severe deficiency, <2 ng/mL), based on information from the National Institutes of Health (NIH) Office of Dietary Supplements Folate Fact Sheet for Health Professionals [12].

Statistical analysis

Data tabulation and analysis were performed using IBM SPSS Statistics for Windows, Version 24.0 (IBM Corp., Armonk, NY). Descriptive statistics, including means and standard deviations (SDs), were computed for the variables. Pearson correlation coefficients were calculated to assess the relationships between these variables.

## Results

The study involved 150 recently identified pregnant females, with a mean age of 29 years (SD = 6.2 years), ranging from 18 to 45 years. The average HCG level among the participants was 4292 mIU/mL (SD = 3,947 mIU/mL), with levels ranging from 98 to 10,000 mIU/mL. The mean serum levels were 17.8 ng/mL (SD = 11.6) for vitamin D, 367 pg/mL (SD = 245) for vitamin B12, and 11.5 ng/mL (SD = 4.6) for folate, as shown in Table 1.

**Table 1:** Means and ranges of vitamin D, vitamin B12, and folate in the study population (N = 150).

Parameter	Range	Mean ± SD
Vitamin D (ng/mL)	3-60	17.8 ± 11.6
Vitamin B12 (pg/mL)	50-1,560	367 ± 245
Folate (ng/mL)	2.1-20	11.5 ± 4.6

The analysis of deficiencies revealed that the prevalence of severe vitamin D deficiency was 92 participants (61.3%) with levels below 20 ng/mL, 39 participants (26%) had insufficient levels (20-29 ng/mL), and only 19 participants (12.7%) possessed sufficient levels (30-100 ng/mL), with no cases of toxicity (>150 ng/mL). Regarding vitamin B12 deficiency (level < 200 pg/mL), 32 participants (21.3%) were found to be deficient, while the remaining 118 participants (78.7%) displayed normal levels (200-900 pg/mL). Folate analysis revealed that 3 participants (2%) exhibited moderate deficiency (2-3 ng/mL), while 14 participants (9.3%) had mild deficiency (3-6 ng/mL), with no instances of severe deficiency (<2 ng/mL), as shown in Table 2.

**Table 2:** Prevalence of deficiencies of vitamin D, vitamin B12, and folate in the study population (N = 150).

Parameter	Categories	Number	Percentage (%)
Vitamin D (ng/mL)	Deficiency (<20)	92	61.3%
	Insufficiency (20-29)	39	26%
	Sufficiency (30-100)	19	12.7%
	Toxicity (>150)	0	0%
Vitamin B12 (pg/mL)	Deficiency (<200)	32	21.3%
	Normal levels (200-900)	118	78.7%
Folate (ng/mL)	Severe deficiency (<2)	0	0%
	Moderate deficiency (2-3)	3	2%
	Mild deficiency (3-6)	14	9.3%
	Normal levels (>6)	133	88.7%

In Table 3, the Pearson correlation coefficients were used to assess the relationships between the study variables. The results indicated that most correlations were weak, highlighting the lack of strong linear relationships between the variables. The strongest correlation observed was a weak negative correlation between age and serum folate levels (-0.18). This meant that as age increased, serum folate levels tended to decrease slightly, although the association was not strong.

**Table 3:** Pearson correlation coefficients for the study variables.

	Age	HCG level	Serum folate	Vitamin B12	Vitamin D
Age	1.00	-0.05	-0.18	-0.06	0.01
HCG level	-0.05	1.00	0.13	-0.18	0.02
Vitamin D	0.01	0.02	0.09	0.13	1.00
Vitamin B12	-0.06	-0.18	0.06	1.00	0.13
Folate	-0.18	0.13	1.00	0.06	0.09

## Discussion

Pregnancy is characterized by an increased demand for essential nutrients, particularly micronutrients, to support fetal development. Micronutrients are crucial for fetal development, significantly impacting neonatal neurological and skeletal development. Deficiencies in these micronutrients before conception can lead to adverse maternal and neonatal health outcomes. Although research on micronutrient deficiencies in the general population has surged in recent years, there remains a lack of localized data on the micronutrient status of pregnant women [13].

Folate is essential for DNA synthesis, repair, and methylation. Folate deficiency during pregnancy is linked to NTDs and other congenital anomalies. Prior research has demonstrated that folate supplementation, both before conception and throughout pregnancy, can significantly reduce the risk of NTDs [14,15]. This study found that 11.3% of participants exhibited folate deficiency, a rate comparable to the 11% reported in similar studies conducted in Canada [15] and among Syrian refugees in Iraq (11.5%) [16].

A comprehensive systematic review of the prevalence of folate deficiency among women of reproductive age revealed that over 20% of women in lower-income economies and fewer than 5% of women in higher-income economies experience folate deficiency [17]. Despite being a lower-income economy, our findings indicate a lower prevalence of folate deficiency among women of reproductive age compared to global rates. However, these rates remain higher than those observed in higher-income countries. This underscores the need to enhance the existing public health program for preconception folate supplementation. While the program provides folate to newly married women, its actual utilization remains uncertain. For instance, a study in Lebanon reported that only 14% of women took folic acid supplements before pregnancy, highlighting the need for greater awareness and educational efforts [7]. Additionally, in northern China, only 36% of women were aware of the benefits of folic acid, and merely 15% took supplements before conception [8].

Vitamin B12 is well known for its role in the formation of red blood cells, neuron myelination, and as a cofactor for enzymes involved in DNA synthesis and repair within the folate pathway. Deficiency in vitamin B12 levels can lead to megaloblastic anemia, neuropsychiatric disorders, and hyperhomocysteinemia, which may negatively affect pregnancy by increasing the risk of preterm labor or low birth weight [14]. In this study, Vitamin B12 deficiency was found in 21.3% of participants, a rate comparable to findings from studies by Sobowale et al. (19%) [18] and Finkelstein et al. (22.6%) [19]. It is important to note that vitamin B12 levels decline as pregnancy progresses due to various hormonal and physiological changes. The prevalence of Vitamin B12 deficiency reported in this study, which pertains to recently identified pregnant women, is expected to increase as pregnancy approaches term. This trend was evident in a study by Ali et al., which found the prevalence of vitamin B12 deficiency across all trimesters to be 73%, with mean vitamin B12 levels declining from 227 pg/mL in the first trimester to 146 pg/mL in the third trimester [16]. These findings suggest that preventive health programs should include vitamin B12 in routine screening and provide supplementation, particularly for vulnerable groups such as those with diets low in animal products, which are the primary sources of Vitamin B12 [8,11].

Vitamin D deficiency during pregnancy has been linked to several adverse outcomes, including gestational diabetes, preeclampsia, and low birth weight, in addition, that is regarded as a risk factor for preterm labor and vaginal bacterial infections [6]. In this study, the prevalence of vitamin D deficiency was 61.3%, with an additional 26% showing vitamin D insufficiency. The prevalence of vitamin D deficiency during pregnancy varies in each country, ranging from 4% to 60% [20]. The high prevalence observed in this study emphasizes the importance of implementing strategies to prevent vitamin D deficiency, which should include education about adequate sun exposure, consumption of vitamin D-rich diets, and taking vitamin D supplements to optimize vitamin D levels during pregnancy [20]. Existing evidence supports that adequate vitamin D levels during pregnancy reduce the risk of preterm labor and assist fetal neurological development [21, 22].

In the correlation analysis, we found that the correlations between deficiencies in folate, vitamin B12, and vitamin D were weakly negative. This means that these deficiencies are independent of each other, highlighting the importance of a panoramic approach to identify and address them. Health professionals caring for pregnant women should conduct a comprehensive assessment of multiple nutrient deficiencies to supplement the deficient nutrients and optimize the nutritional status of pregnant women, thereby improving pregnancy outcomes. Preventive health programs should aim to increase public awareness about the benefits of improving nutritional levels before and during pregnancy. Additionally, food fortification with essential vitamins and trace minerals remains an important public health strategy to prevent these deficiencies [22].

Limitations of this study include the small sample size, which may affect the generalizability of the results. It is important to consider larger-scale studies to confirm the findings of this study as well as, the presence of another healthy group is important for comparison to know the prevalence of the deficiency of these nutrients in the general population. Future studies should also consider including additional measurements like calcium and phosphorus levels, which are important for exploring vitamin D deficiency, and homocysteine levels, which are important in vitamin B12 deficiency. 

## Conclusions

In conclusion, vitamin D deficiency was high, while deficiencies of folate and vitamin B12 were comparable to worldwide estimates. These findings focus attention on the existence of nutritional deficiencies in newly diagnosed pregnant women and urge for preventive health interventions to correct these deficiencies to achieve the best pregnancy outcomes from maternal and fecal perspectives. Ensuring adequate levels of these essential vitamins is crucial for preventing adverse pregnancy outcomes, such as neural tube defects, megaloblastic anemia, and impaired fetal skeletal development. Our research calls for healthcare professionals, policymakers, and preventive health program managers to prioritize micronutrient screening and intervention programs for pregnant women to improve pregnancy outcomes and promote long-term health benefits for both mothers and their children.
